# Behçet’s disease in HLA-B*51 negative Germans and Turks shows association with HLA-Bw4-80I

**DOI:** 10.1186/ar4569

**Published:** 2014-05-26

**Authors:** Alexandr Borisovich Kuranov, Ina Kötter, Jörg Christoph Henes, Saule Tleubaevna Abisheva, Ingeborg Steiert, Florian Riewerts, Kuvat Temirgalievich Momynaliev, Claudia Anna Müller

**Affiliations:** 1 Department of Internal Medicine II, Section for Transplantation Immunology and Immunohematology, University of Tübingen, Waldhoernle Str. 22, 72072 Tübingen, Germany; 2 Interdisciplinary Centre for Rheumatology Stuttgart (ZIRS), Stuttgart, Germany; 3 Centre for Interdisciplinary Clinical Immunology, Rheumatology and Auto-inflammatory Diseases-INDIRA and Department of Internal Medicine II, University of Tübingen, Tübingen, Germany; 4 Scientific Research Institute of Traumatology and Orthopedics, Astana, Kazakhstan; 5 National Center for Biotechnology, Astana, Kazakhstan

## Abstract

**Introduction:**

Behçet’s disease (BD) as systemic vasculitis of unknown etiology is associated with HLA-B*51 in European and Asian populations. HLA-A*26 was claimed as an additional BD susceptibility marker in Japanese and Greek patients. This study was performed to test for HLA associations in HLA-B*51 negative German and Turkish BD populations.

**Methods:**

In total, 65 German and 46 Turkish patients lacking HLA-B*51 were analyzed in comparison to healthy HLA-B*51 negative Germans (n = 1500) and Turks (n = 130). HLA-A/B genotypes were determined by SSOP. P-values with correction for multiple testing (p_c_), *χ2****-***test and odds ratio (OR) were used for statistical evaluation.

**Results:**

HLA-A*26 was significantly more frequent in HLA-B*51^−^ German patients [p_c_ = 0.0076, OR = 3.23, 95% CI 1.63 to 6.39] than in respective controls. HLA-A*26 was also elevated in a smaller group of Turkish patients versus the controls. Significant association of HLA-Bw4 with isoleucine at amino-acid position 80 (HLA-Bw4-80I) was found in the HLA-B*51^−^ German cohort of BD patients [p_c_ = 0.0042, OR = 2.35, 95% CI 1.41 to 3.93) and in the Turkish patients in comparison to the respective controls [*p* = 0.025, OR = 2.17, 95% CI 1.09 to 4.31]. On the contrary, HLA-Bw4-80 T was reduced in both HLA-B*51^−^ BD patient cohorts.

**Conclusions:**

The study shows a significant association of HLA-Bw4-80I present on HLA-B*51 as well as on other B-locus molecules with BD. This indicates that distinctive Bw4 epitopes on HLA-B locus molecules could play a role in BD pathogenesis. The study also indicates an association with HLA-A*26 in German and Turkish BD patients as a genetic risk factor independent of HLA-B*51.

## Introduction

Behçet’s disease (BD) is a chronic relapsing multisystem disorder of still unknown etiology with histopathologic features of a leucocytoclastic vasculitis. It frequently manifests with recurrent oral and genital aphthosis, ocular involvement, skin inflammation, and arthritis, but also can affect many other organs. As a pathognomonic test for BD is still lacking, International Criteria for BD (ICBD) [1] based on characteristic clinical symptoms have been developed for standardized diagnosis. BD today occurs throughout the world, but was classically only seen with a high prevalence in countries along the old Silk routes leading from the Mediterranean via the Middle East to Japan. In the native German population, BD is diagnosed as a rare disease with an estimated prevalence of 0.6 cases per 100,000 inhabitants [2,3] and clinical manifestations similar to other ethnic groups.

An increased risk of BD has been noted in siblings of BD patients for a long time and interpreted as a sign of genetic susceptibility. In 1978, Ohno *et al*. [4] first described the human leucocyte antigen gene (HLA)-B*51 (B*51) as a major genetic risk factor for BD, which was subsequently confirmed in many other ethnic populations tested with odds ratios (ORs) up to 5.78 [5]. Up to now it is still unknown, whether HLA-B*51 as a molecule itself or other linked genes within the HLA system primarily contribute to BD susceptibility [6]. Many association studies of BD in different ethnic groups have shown that only 60 to 75% of the cases carry HLA-B*51. Also, in the German population, about 25% of the BD patients are at least HLA-B*51-negative [7]. These observations suggest additional BD susceptibility genes. Recently, advanced molecular linkage studies proposed MICA and/or HLA-A*26:01 as genetic risk factors independent of HLA-B*51, particularly in Japanese [8], Taiwanese [9] and Greek [10] BD patients. Further analyses suggested that other HLA molecules, particularly of the HLA-B locus, might also be associated with BD [11]. Recent advances in sequencing technology and genome-wide analyses provided more evidence for additional genetic associations between BD and multiple single nucleotide polymorphism (SNP) variants within the HLA-system. This pertains to PSORS1C1 polymorphism, variants upstream of HLA-F-AS1 and to HLA-C*16:02 [12]. Different loci outside of the major histocompatability complex (MHC), such as IL-10, IL-23R, CCR1, STAT4, MEFV, toll-like receptor (TLR)4, which regulate inflammation or are involved in innate immunity, have been also claimed to be linked to BD [13]. Furthermore, recent reports about an association of BD with ERAP1 and KLRC4 implicated altered peptide-MHC class I interactions with natural killer (NK) cell receptors which could influence NK-cell activity in the pathogenesis of the disease [11].

This study was performed to validate and test for HLA-A and further HLA-B associations in German BD patients as well as in a cohort of Turkish patients with long-standing German residency. Both BD patient groups and the respective healthy control groups were stratified according to their lack of an HLA-B*51 phenotype. HLA-B*51-negative BD patients were compared with respective healthy control groups for further HLA-associations independent of HLA-B*51.

## Methods

### Patients and controls

German (n = 65) and Turkish (n = 46) unrelated BD patients, who presented themselves at the outpatient clinic of the University Hospital of Tübingen (Germany) during the previous five years, were included in this study. The patients were selected from a larger group of outpatients with BD as HLA-B*51-negative cases after routine HLA-typing. In all patients, BD was diagnosed according to the International Criteria for BD (ICBD) [1]. The Turkish (T) and German (G) BD patients presented with the following clinical symptoms: oral aphthae (n (T/G) = 34/49), genital ulcers (n (T/G) = 8/25), erythema nodosum (n (T/G) = 21/16), papulopustules (n (T/G) = 23/35), folliculitis (n (T/G) = 1/5), arthritis (n (T/G) = 17/25), cerebral attack (n (T/G) = 1/3), ocular manifestation (n (T/G) = 16/25) and venous thrombosis (n (T/G) = 7/7). Local panels of 1,500 healthy German and 130 Turkish individuals negative for HLA-B*51 were used for comparison of HLA-allele frequencies in both patient populations. The study was approved by the *Ethik-Kommission* of the Medical Faculty, University of Tübingen (project 225/2007BO2). Blood samples were collected after written informed consent of all participants for their enrollment.

### HLA genotyping

Genomic DNA was isolated with the use of QIAamp Blood Mini kits (Qiagen N.V. Hilden, Germany) from 5 ml EDTA blood samples drawn from each patient, as well as from the controls, after informed consent. HLA-A und -B genotypes were determined by LUMINEX technology with the use of the LABType (®) SSO typing kits (One Lambda, Inc., Canoga Park, CA, USA). HLA Fusion™ Software Version 2.0 (One Lambda Inc.) was used for data analysis.

### Statistical analysis

Allele (determinant) frequencies were estimated by direct counting. The 2 × 2 contingency tables for each individual allele were used, where n refers to the number of alleles detected in the population. The strength of the association was estimated by an OR of risk and 95% CI. We used χ^2^-analysis, *P*-values, and ORs with 95% CI for statistical evaluation of HLA-allele differences between BD patients and controls. The *P*-values (corrected *P* (*P*_c_-value)) were corrected by multiplying the number of observed alleles at the locus. The significance level was set at *P*_c_ <0.05 for all analyses. All *P*-values were derived from a two-sided test. All statistical calculations were computed using the Epi Info™ Version 7.0.8.3 (Clifton Rd, Atlanta, GA, USA). In addition, two-locus haplotypes were generated by Arlequin (v3.11) software using the allele status at the HLA-A and B locus. The Bonferroni correction was applied to the *P*-values [14,15].

## Results

### HLA-A associations in B*51 negative BD patients

To evaluate the influence of an HLA-A-linked second risk locus, independently associated with BD of HLA-B*51, distribution of HLA-A alleles was evaluated in BD. We analyzed 65 German BD patients who were typed negative for HLA-B*51 and compared them to 1,500 HLA-B*51-negative healthy German controls (see Additional file 1: Table S1). HLA-A*26 was significantly more frequent in the German patients than in the respective healthy controls (11 (16.9%) patients versus 89 (5.9%) healthy Germans, *P*_c_ = 0.0076, OR = 3.23, 95% CI 1.63 to 6.39), as it had been previously reported for Japanese [8], Taiwanese [9] and Greek [10] patients. In addition, a tendency for a negative association with HLA-A*03 was observed in the German BD patients in comparison to the healthy control group (11 (16.9%) patients versus 451 (30.1%) healthy German controls, *P* = 0.022, *P*_c_-value not significant, OR = 0.47, 95% CI 0.25 to 0.91). In contrast to other studies, HLA-A*26 was also elevated in the HLA-B*51-negative Turkish BD patients (5 (10.9%) patients versus 1 (0.8%) healthy Turks, *P*_c_ = 0.019, OR = 15.73, 95% CI 1.78 to 138.56), however, significance should be confirmed in a larger patient group. HLA-A*32 also showed a slight elevation in the Turkish BD patients. No other significant HLA-A differences were seen in German and Turkish BD patients in comparison to the respective healthy controls.

### HLA-B associations in B*51-negative BD patients

Elevated frequencies of various HLA-B locus alleles, such as B*39, B*45, B*55 and B*57 (see Additional file 1: Table S2), were observed in the German BD patients in comparison to the controls. On the contrary, HLA-B*08 and HLA-B*40 alleles seemed to be negatively associated with BD in Germans. In Turks, only HLA-B*57 was significantly and consistently raised in the patient group. In contrast, HLA-B*18 showed a tendency of a negative association with BD in Turks.

These diverse HLA-B associations in HLA-B*51-negative German and Turkish patients as well as previous experimental hints for an involvement of NK cells in the pathogenesis of BD raised the question, could HLA as a ligand of NK cell receptors, in particular HLA-Bw4 as a public determinant of HLA-B locus antigens, play a role in genetic susceptibility to BD? In this regard, it was of interest that HLA-B*51 strongly associated with BD belongs to the HLA-B locus alleles coding for a Bw4 determinant with isoleucine at amino acid position 80. Analysis of the HLA-Bw4 determinant on HLA-B alleles showed only a slight elevation in the German patient group in comparison to healthy controls (see Additional file 1: Table S3). This was also seen for Bw4 as a determinant encoded by some HLA-A alleles (A*23, A*24, A*25, A*32) or in a combined analysis of Bw4-positive HLA-B and -A alleles (see Additional file 1: Table S3). Bw4 homozygotes tended to be increased, whereas Bw6 homozygotes seemed to be slightly decreased in the German BD patients. Similar results were obtained for B*51-negative Turkish patients. The Bw4 epitope is characterized by the NIALR sequence motif of the polymorphic amino acid positions 77, 80 to 83 in the α_1_ helix of the HLA molecule [16]. Several studies have shown that variant Bw4 HLA-B allotypes with isoleucine or threonine at position 80 can be discriminated by NK cells and manifest associations with different clinical diseases. Analysis of these Bw4 HLA-B allotypes in the B*51-negative BD patients revealed a significant association of BD with presence of Bw4-80I, but not of Bw4-80 T in the German groups (Bw4-80I *P* = 0.0007, *P*_c_ = 0.0042, OR = 2.35, 95% CI 1.41 to 3.93) (see Additional file 1: Table S4). The same association was found in the Turkish BD patient group, but with a significance level not as robust as in the German patients after correction for multiple testing. Interestingly, when we excluded all BD patients carrying Bw4^+^ HLA-B alleles, an association of BD to HLA-A*26 was no longer seen. Strong linkage disequilibrium (LD) of HLA-A*26 with the HLA-Bw4-80I allele B*49 (DÂ€ʹ=1.0; P = 0.001) (see Additional file 1: Table S5) was observed in German patients, but not in the respective controls. This indicated that the observed HLA-A*26 association could be a result of LD with Bw4-80I-positive HLA-B alleles. No significant specific LD of HLA-A*26 with HLA-B alleles was observed in the Turkish patients and healthy controls. When the HLA-B*51-negative patient and control groups were stratified for presence or absence of HLA-A*26, the association of HLA-Bw4-80I with BD still held true in the German patients (see Additional file 1: Table S6) Only a weak association of HLA-Bw4-80I with BD was calculated from a similar regression analysis of the Turkish cohorts.

## Discussion

As in other ethnicities, HLA-B*51 has been previously shown to be associated with BD in Germans, where this disease rarely occurs in the native population. In this study, new and additional HLA-linked associations were discovered in German BD patients who were recruited as HLA-B*51 non-carriers. First, HLA-A*26 was found to be strongly associated with BD in Germans, as it had been reported for Japanese [8], Greek [10] and Taiwanese [9] cohorts. Additionally, our results clearly identified the HLA-Bw4 epitope with isoleucine at position 80 as a possible major genetic susceptibility determinant of BD in Germans. Comparative analysis of Turkish resident HLA-B*51 non-carriers in Germany also indicated HLA-A*26 as a BD-associated susceptibility marker and showed a significant, but less robust linkage with the HLA-Bw4-80I epitope.

HLA-A*26 is one of the less frequent HLA-A alleles in Germans, with a phenotype frequency of 5.9% in healthy controls. It was clearly increased in the German patient group lacking the HLA-B*51 allele with the strongest BD association of all seen in the analyzed HLA alleles. This finding, therefore, supports results of previous studies, which suggested that the HLA-A*26 allele itself represents an additional BD susceptibility gene independent of HLA-B*51. LD analysis, however, indicated that the HLA-A*26 association could be a result of LD with the Bw4-80I-positive HLA-B allele B*49 in German patients. The phenotype frequency of HLA-A*26 appeared to be also increased in the Turkish patients, but not enough HLA-B*51 non-carrier controls could be recruited to establish a statistically safe association. So far, the role of HLA-A*26 in the pathogenesis of BD still remains incompletely defined. HLA-A*26 has been linked to acute Graft-versus-host disease (GVHD) after allogeneic stem cell transplantation [17], to lymphoproliferative disorders after solid organ grafts [18], as well as to specific forms of psoriatic arthritis [19] as a sign for a pathogenetic role also in other clinical diseases. At the molecular level HLA-A*26 belongs to the HLA-A molecules that bind with reduced affinity to the inhibitory leucocyte immunoglobulin-like receptors (LILR) B1 on myeloid cells, including dendritic cells, as well as on other different immune cells that are involved in innate immunity and inflammation [20,21]. Thus, for further insight into the pathogenesis of BD, it could be of relevance to experimentally clarify if such interactions influence the activity of professional antigen-presenting cells, innate immune activation or inflammation in BD. In this respect, HLA-A*03, which on the contrary binds with stronger affinity than HLA-A*26 to LILRB1 [20] and also ligates to the NK cell receptor KIR3DL2 [22], could be of interest as a potential protective allele with an observed negative association to BD and thus should be investigated in further larger patient cohorts.

HLA-B locus alleles can be divided in two groups according to their expression of the allelic determinants Bw4 and Bw6 [23,24]. The Bw4 epitope with the common sequence NIALR at position 77 to 83 of the α_1_ helix of the HLA-B heavy chain has been shown to function as the binding site of the killer immunoglobulin-like receptors (KIR) KIR3DL1 on NK cells, with influences on NK and T-cell activation in innate and adaptive immune responses [25]. So far, in several studies, abnormalities in circulating NK cells have been reported and shown to correlate with disease activity or response to therapy [26,27]. Therefore, we analyzed frequency of the Bw4 epitope either expressed on HLA-A or HLA-B or on allotypes of both loci in the German and Turkish patient groups of HLA-B*51 non-carriers. However, no significant differences between BD patients and controls were found in Germans and Turks. The HLA-Bw4 epitope exists on HLA-B and some HLA-A proteins in at least eight variant subtypes with differential binding activity to KIR3DL1, in particular for the dimorphic Bw4 allotypes with either isoleucine (Bw4-80I) or threonine (Bw4-80 T) at amino acid position 80 [16]. Interestingly, for the first time we observed that the HLA-B/Bw4-80I, but not the Bw4-80 T variant was significantly associated with BD in a German and to a weaker statistical power also in a Turkish patient cohort of HLA-B*51 non-carriers. HLA-B*51, which was previously shown to be strongly associated with BD also codes for the Bw4-80I epitope. As such, the HLA-B*51 HLA-association of BD does not contradict our hypothesis that HLA-Bw4-80I could play a role in pathogenesis of the disease through the control of NK-/T-cell interactions with myeloid cells via ligation with KIR3DL allotypes. In a previous analysis by Middleton *et al*. [28] no association between KIR genes and BD in B*51-positive/negative or Bw4-positive/negative patients had been observed. This supports our observation that not all Bw4 determinants, but only Bw4-80-I ligands might be associated with BD. KIR3DL1 is a highly polymorphic locus with alleles coding for NK cell inhibiting and activating molecules. According to Norman *et al*. [29] all KIR3DL1 variants can be grouped into one of three families with sequence variation at specific amino acid positions maintained by balancing selection through human evolution. DX9, a monoclonal antibody against KIR3DL1 molecules appears to differentiate the products of these three lineages [30]. In a recent analysis, it was used to stain lymphocyte and NK cells in BD, but did not detect differences in the expression of KIR3DL1 in patients and controls [31]. Nevertheless, like in other diseases with evidence of a pathogenic role of the KIR3DL1/Bw4 interaction [32], it may also be relevant in BD to precisely determine the specific KIR3DL1 subtypes in order to detect mutual influences of NK cell receptors together with Bw4-80I on the pathogenesis of the disease.

In addition, it has been shown that pathogen- or stress-derived peptides presented by the HLA-B molecule could alter the binding face of the Bw4 epitope for KIR interaction [33,34]. In this respect, it may be of interest to note that the expression of the Bw4 epitope on granulocytes significantly varied from the Bw4 epitope on lymphocytes in BD patients, but not in controls (manuscript in preparation). Since KIR expression is also found on different T-cell subtypes, variant expression could also be critical for disease development in cell types other than NK cells, and it will thus be a challenge for future analysis to identify effector and target cells with altered KIR/Bw4 interaction in BD.

## Conclusion

The study suggests for the first time that HLA-B*51 together with other HLA-Bw4-80I-positive HLA-B molecules might play an important role as a T-cell as well as an NK-cell ligand in the pathogenesis of BD. The observed association of HLA-A*26 with BD, particularly in the German patients, appeared to also depend on the linkage to a Bw4-80I-positive HLA-B allele and thus, did not unequivocally point to a second HLA-A-linked susceptibility locus of BD.

## Abbreviations

BD: Behçet’s disease; HLA: human leukocyte antigen; IL: interleukin; KIR: killer immunoglobulin-like receptor; LD: linkage disequilibrium; MHC: major histocompatability complex; NK: natural killer; OR: odds ratio; *P*_c_: Bonferroni-corrected *P*-value.

## Competing interests

The authors declare that they have no competing interests.

## Authors’ contributions

ABK performed all the experimental work, data collection and analysis of the paper and wrote the manuscript. IK was involved in coordinating and supervising data collection. JCH is a Consultant Rheumatologist and was involved in patient recruitment for the study. STA was involved in manuscript preparation. IS and FR contributed to sample collection and preparation. KTM read the manuscript and guided the experimental and analytical part. CAM, as the project leader, designed the study, reviewed the data and manuscript writing. All authors have read and approved the manuscript for publication.

## Supplementary Material

Additional file 1: Table S1 Frequency of human leukocyte antigen (HLA)-A alleles in B*5-negative German and Turkish Behçet’s disease (BD) patients in comparison to healthy controls. **Table S2.** Frequency of HLA-B alleles in B*51-negative BD patients and controls. **Table S3.** Frequency of the Bw4 determinant in B*51-negative BD patients and controls. **Table S4.** Frequency of Bw4 determinants (Bw4-80I and Bw4-80 T) in B*51-negative BD patients and controls. **Table S5.** Linkage disequilibrium of HLA-A*26 with HLA-B locus alleles (two-loci haplotype frequencies) in patients and controls. **Table S6.** Frequency of Bw4 determinants (Bw4-80I versus Bw4-80 T) in A*26-negative/B*51-negative BD patients and controls. Click here for file
