# Supplementary material for: Behçet’s disease in HLA-B*51 negative Germans and Turks shows association with HLA-Bw4-80I
Source: Arthritis Res Ther. 2014 May 26;16(3):R116. doi: 10.1186/ar4569 (PMC4075409; doi:10.1186/ar4569)
Supplement: Additional file 1: Table S1 — Frequency of human leukocyte antigen (HLA)-A alleles in B*5-negative German and Turkish Behçet’s disease (BD) patients in comparison to healthy controls. Table S2. Frequency of HLA-B alleles in B*51-negative BD patients and controls. Table S3. Frequency of the Bw4 determinant in B*51-negative BD patients and controls. Table S4. Frequency of Bw4 determinants (Bw4-80I and Bw4-80 T) in B*51-negative BD patients and controls. Table S5. Linkage disequilibrium of HLA-A*26 with HLA-B locus alleles (two-loci haplotype frequencies) in patients and controls. Table S6. Frequency of Bw4 determinants (Bw4-80I versus Bw4-80 T) in A*26-negative/B*51-negative BD patients and controls. [file ar4569-S1.pdf]

Table 1 Frequency of HLA-A alleles in B*51 negative German and Turkish BD patients in comparison to healthy controls.

HLA-A allele	Germans BD (%) (N=65)	Germans Controls (%) (N=1500)	*χ2*	*p Value*	*p_c Value*	*OR (95% CI)*	Turks BD (%) (N=46)	Turks Controls (%) (N=130)	*χ2*	*p Value*	*p_c Value*	*OR (95% CI)*
*01	18 (27.7)	485 (32.3)	0.62	NS	NS	0.80 (0.46 to 1.39)	11 (23.9)	23 (17.7)	0.84	NS	NS	1.46 (0.65 to 3.30)
*02	37 (56.9)	831 (55.4)	0.92	NS	NS	1.06 (0.64 to 1.76)	15 (32.6)	55 (42.3)	1.33	NS	NS	0.66 (0.33 to 1.34)
*03	11 (16.9)	451 (30.1)	5.17	0.022	NS	0.47 (0.25 to 0.91)	9 (19.6)	26 (20.0)	0.004	NS	NS	0.97 (0.42 to 2.27)
*11	4 (6.2)	134 (8.9)	0.60	NS	NS	0.67 (0.24 to 1.87)	7 (15.2)	24 (18.5)	0.25	NS	NS	0.79 (0.32 to 1.99)
*23	0 (0.0)	60 (4.0)	-	-	-	-	3 (6.5)	6 (4.6)	0.25	NS	NS	1.44 (0.35 to 6.01)
*24	14 (21.5)	273 (18.2)	0.46	NS	NS	1.23 (0.67 to 2.26)	13 (28.3)	41 (31.5)	0.17	NS	NS	0.86 (0.41 to 1.79)
*25	6 (9.2)	64 (4.3)	3.59	NS	NS	2.28 (0.95 to 5.48)	0 (0.0)	0 (0.0)	-	-	-	-
*26	11 (16.9)	89 (5.9)	12.58	0.0004	0.0076	3.23 (1.63 to 6.39)	5 (10.9)	1 (0.8)	10.53	0.001	0.019	15.73 (1.78 to 138.56)
*29	2 (3.1)	68 (4.5)	0.31	NS	NS	0.67 (0.16 to 2.79)	1 (2.2)	9 (6.9)	1.43	NS	NS	0.30 (0.04 to 2.43)
*30	1 (1.5)	41 (2.7)	0.34	NS	NS	0.57 (0.08 to 4.11)	4 (8.7)	7 (5.4)	0.64	NS	NS	1.67 (0.47 to 6.00)
*31	2 (3.1)	42 (2.8)	0.03	NS	NS	1.13 (0.26 to 4.77)	4 (8.7)	8 (6.1)	0.35	NS	NS	1.45 (0.42 to 5.01)
*32	2 (3.1)	87 (5.8)	0.86	NS	NS	0.52 (0.12 to 2.14)	7 (15.2)	7 (5.4)	4.49	0.034	NS	3.15 (1.04 to 9.55)
*33	0 (0.0)	31 (2.1)	-	-	-	-	3 (6.5)	13 (10.0)	0.49	NS	NS	0.62 (0.17 to 2.31)
*34	0 (0.0)	3 (0.2)	-	-	-	-	0 (0.0)	0 (0.0)	-	-	-	-
*36	0 (0.0)	0 (0.0)	-	-	-	-	1 (2.2)	7 (5.4)	-	-	-	-
*66	0 (0.0)	10 (0.7)	-	-	-	-	0 (0.0)	2 (1.5)	-	-	-	-
*68	6 (9.2)	102 (6.8)	0.57	NS	NS	1.39 (0.59 to 3.31)	5 (10.9)	14 (10.8)	0.0004	NS	NS	1.01 (0.34 to 2.98)
*69	0 (0.0)	0 (0.0)	-	-	-	-	1 (2.2)	3 (2.3)	0.0027	NS	NS	0.94 (0.09 to 9.27)
*74	1 (1.5)	0 (0.0)	-	-	-	-	0 (0.0)	0 (0.0)	-	-	-	-

BD, Behçet’s disease; OR, odds ratio; pc, Bonferroni corrected p value.

Table 2 Frequency of HLA-B alleles in B*51 negative BD patients and controls.

HLA-B allele	Germans BD (%) (N=87)	Controls (%) (N=1500)	*χ2*	*p Value*	*p_c Value*	*OR(95% CI)*	Turks BD (%) (N=60)	Controls (%) (N=130)	*χ2*	*p Value*	*p_c Value*	*OR(95% CI)*
*07	22 (25.3)	450 (30.0)	0.87	NS	NS	0.79 (0.48 to 1.30)	6 (10.0)	9 (6.9)	0.53	NS	NS	1.49 (0.51 to 4.41)
*08	10 (11.5)	333 (22.2)	5.56	0.018	0.05	0.46 (0.23 to 0.89)	5 (8.3)	11 (8.5)	0.001	NS	NS	0.98 (0.33 to 2.97)
*13	4 (4.6)	99 (6.6)	0.54	NS	NS	0.68 (0.25 to 1.90)	2 (3.3)	9 (6.9)	0.97	NS	NS	0.46 (0.09 to 2.21)
*14	3 (3.5)	23 (1.5)	1.87	NS	NS	2.29 (0.68 to 7.79)	0 (0.0)	6 (4.6)	-	-	-	-
*15	14 (16.1)	213 (14.2)	0.24	NS	NS	1.16 (0.64 to 2.09)	5 (8.3)	17 (13.1)	0.90	NS	NS	0.60 (0.21 to 1.72)
*18	8 (9.2)	157 (10.5)	0.14	NS	NS	0.87 (0.41 to 1.83)	1 (1.7)	18 (13.8)	6.77	0.009	NS	0.11 (0.01 to 0.81)
*27	10 (11.5)	116 (7.7)	1.59	NS	NS	1.54 (0.78 to 3.07)	2 (3.3)	16 (12.3)	3.86	NS	NS	0.25 (0.05 to 1.11)
*35	15 (17.2)	285 (19.0)	0.17	NS	NS	0.89 (0.50 to 1.57)	20 (33.3)	47 (36.2)	0.14	NS	NS	0.88 (0.46 to 1.68)
*37	1 (1.1)	44 (2.9)	0.95	NS	NS	0.38 (0.05 to 2.83)	1 (1.7)	2 (1.5)	0.004	NS	NS	1.08 (0.09 to 12.20)
*38	0 (0.0)	66 (4.4)	-	-	-	-	6 (10.0)	11 (8.5)	0.12	NS	NS	1.20 (0.42 to 3.42)
*39	10 (11.5)	54 (3.6)	13.24	0.0003	0.009	3.48 (1.71 to 7.09)	2 (3.3)	5 (3.8)	0.03	NS	NS	0.86 (0.16 to 4.58)
*40	8 (9.2)	277 (18.5)	4.80	0.028	NS	0.45 (0.21 to 0.94)	6 (10.0)	20 (15.4)	1.01	NS	NS	0.63 (0.24 to 1.67)
*41	4 (4.6)	35 (2.3)	1.76	NS	NS	2.02 (0.70 to 5.81)	7 (11.7)	7 (5.4)	2.37	NS	NS	2.32 (0.76 to 6.94)
*42	0 (0.0)	0 (0.0)	-	-	-	-	0 (0.0)	1 (0.8)	-	-	-	-
*44	21 (24.1)	378 (25.2)	0.05	NS	NS	0.86 (0.47 to 1.59)	11 (18.3)	20 (15.4)	0.26	NS	NS	1.23 (0.55 to 2.77)
*45	4 (4.6)	8 (0.5)	18.10	0.00002	0.0006	8.99 (2.65 to 30.46)	1 (1.7)	2 (1.5)	0.004	NS	NS	1.08 (0.09 to 12.20)
*46	0 (0.0)	0 (0.0)	-	-	-	-	0 (0.0)	2 (1.5)	-	-	-	-
*47	1 (1.1)	21 (1.4)	0.04	NS	NS	0.82 (0.11 to 6.16)	0 (0.0)	0 (0.0)	-	-	-	-
*48	0 (0.0)	0 (0.0)	-	-	-	-	0 (0.0)	2 (1.5)	-	-	-	-
*49	2 (2.3)	36 (2.4)	0.004	NS	NS	0.96 (0.23 to 4.04)	5 (8.3)	4 (3.1)	2.51	NS	NS	2.81 (0.45 to 17.53)
*50	4 (4.6)	34 (2.3)	1.91	NS	NS	2.08 (0.72 to 5.99)	3 (5.0)	8 (6.2)	0.10	NS	NS	0.80 (0.21 to 3.14)
*52	3 (3.5)	42 (2.8)	0.13	NS	NS	1.24 (0.38 to 4.08)	5 (8.3)	12 (9.2)	0.04	NS	NS	0.89 (0.30 to 2.66)
*53	0 (0.0)	3 (0.2)	-	-	-	-	4 (6.7)	4 (3.1)	1.31	NS	NS	2.25 (0.54 to 9.32)
*55	2 (2.3)	1 (0.1)	21.72	0.000004	0.0001	35.3 (3.17 to 392.8)	8 (13.3)	10 (7.7)	1.52	NS	NS	1.85 (0.69 to 4.94)
*56	0 (0.0)	0 (0.0)	-	-	-	-	0 (0.0)	1 (0.8)	-	-	-	-
*57	17 (19.5)	114 (7.6)	15.48	0.00008	0.002	2.95 (1.68 to 5.19)	8 (13.3)	3 (2.3)	9.14	0.0002	0.006	6.51 (1.66 to 25.52)
*58	2 (2.3)	21 (1.4)	0.47	NS	NS	1.65 (0.38 to 7.18)	4 (6.7)	4 (3.1)	1.31	NS	NS	2.25 (0.54 to 9.32)
*62	1 (1.1)	0 (0.0)	-	-	-	-	0 (0.0)	0 (0.0)	-	-	-	-
*73	0 (0.0)	3 (0.2)	-	-	-	-	0 (0.0)	0 (0.0)	-	-	-	-

BD, Behçet’s disease; OR, odds ratio; pc, Bonferroni corrected p value.

Table 3 Frequency of the Bw4 determinant in B*51 negative BD patients and controls.

	Germans					Turks				
	BD (%) (N=65)	Controls (%) (N=1500)	χ2	*p Value*	*OR (95% CI)*	BD (%) (N=46)	Controls (%) (N=130)	χ2	*p Value*	*OR (95% CI)*
HLA-A (Bw4)	22 (33.8)	423 (28.2)	0.98	NS	1.30 (0.77 to 2.20)	20 (43.5)	57 (43.8)	0.002	NS	0.99 (0.50 to 1.94)
HLA-B (Bw4)	39 (60.0)	822 (54.8)	0.68	NS	1.23 (0.74 to 2.05)	28 (60.9)	75 (57.7)	0.14	NS	1.11 (0.56 to 2.20)
HLA-A/B (Bw4)	46 (70.8)	1007 (67.1)	0.37	NS	1.10 (0.64 to 1.88)	38 (82.6)	98 (75.4)	1.01	NS	1.55 (0.66 to 3.67)
B/Bw4 homo	10 (15.4)	160 (10.6)	1.43	NS	1.52 (0.76 to 3.05)	9 (19.6)	13 (10.0)	2.84	NS	2.19 (0.87 to 5.53)
B/Bw4/Bw6 hetero	29 (44.6)	662 (44.1)	0.01	NS	1.02 (0.62 to 1.68)	19 (41.3)	62 (47.7)	0.56	NS	0.75 (0.38 to 1.48)
B/Bw6 homo	26 (40.0)	678 (45.2)	0.68	NS	0.81 (0.49 to 1.34)	18 (39.1)	55 (42.3)	0.14	NS	0.90 (0.46 to 1.80)

BD, Behçet's disease; OR, odds ratio; pc, Bonferroni corrected p value.

Table 4 Frequency of Bw4 determinants (Bw4-80I and Bw4-80T) in B*51 negative BD patients and controls.

	Germans						Turks					
	BD (%) (N=65)	Controls (%) (N=1500)	χ2	*p Value*	*p_c Value*	*OR (95% CI)*	BD (%) (N=46)	Controls (%) (N=130)	χ2	*p Value*	*p_c Value*	*OR (95% CI)*
Bw4-80I	26 (40.0)	331 (22.1)	11.38	0.0007	0.0042	2.35 (1.41 to 3.93)	23 (50.0)	41 (31.5)	5.00	0.025	NS	2.17 (1.09 to 4.31)
Bw4-80T	23 (35.4)	605 (40.3)	0.64	0.43	NS	0.81 (0.48 to 1.36)	12 (26.1)	44 (33.8)	0.94	0.33	NS	0.69 (0.33 to 1.46)

BD, Behçet's disease; OR, odds ratio; pc, Bonferroni corrected p value

Table 5 Linkage disequilibrium of HLA-A*26 with HLA-B locus alleles (two-loci haplotype frequencies) in patients and controls.

Turkish patients (N=46)	χ^2	*p Value*	*D´*	Turkish controls (N=130)	χ^2	*p Value*	*D´*
HLA-A*26 –HLA-B*40	-	-	-	HLA-A*26 –HLA-B*40	7.45	0.01	0.27
HLA-A*26 –HLA-B*38	-	-	-	HLA-A*26 –HLA-B*38	7.45	0.01	0.19
German patients (N=65)	χ^2	*p Value*	*D´*	German controls (N=1500)	χ^2	*p Value*	*D´*
HLA-A*26 –HLA-B*40	6.68	0.0098	0.34	HLA-A*26 –HLA-B*40	33.31	0.001	0.12
HLA-A*26 –HLA-B*49	10.90	0.0010	1.00	HLA-A*26 –HLA-B*27	6.2	0.01	0.05

LD measures are presented in D´, a standardised measure that ranges from 0 to 1.

Table 6 Frequency of Bw4 determinants (Bw4-80I versus Bw4-80T) in A*26 negative/B*51 negative BD patients and controls.

	Germans						Turks					
	BD (%) (N=54)	Controls (%) (N=1411)	*χ2*	*p Value*	*p_c Value*	*OR (95% CI)*	BD (%) (N=41)	Controls (%) (N=129)	*χ2*	*p Value*	*p_c Value*	*OR (95% CI)*
Bw4-80I	21 (38.9)	293 (20.8)	10.1	0.001	0.006	2.42 (1.38 to 4.26)	21 (51.2)	40 (31.0)	5.52	0.018	NS	2.34 (1.14 to 4.78)
Bw4-80T	21 (38.9)	567 (40.2)	0.03	0.87	NS	1.05 (0.60 to 1.83)	12 (29.3)	43 (33.3)	0.23	0.62	NS	0.83 (0.38 to 1.78)

BD, Behçet’s disease; OR, odds ratio; pc, Bonferroni corrected p value
